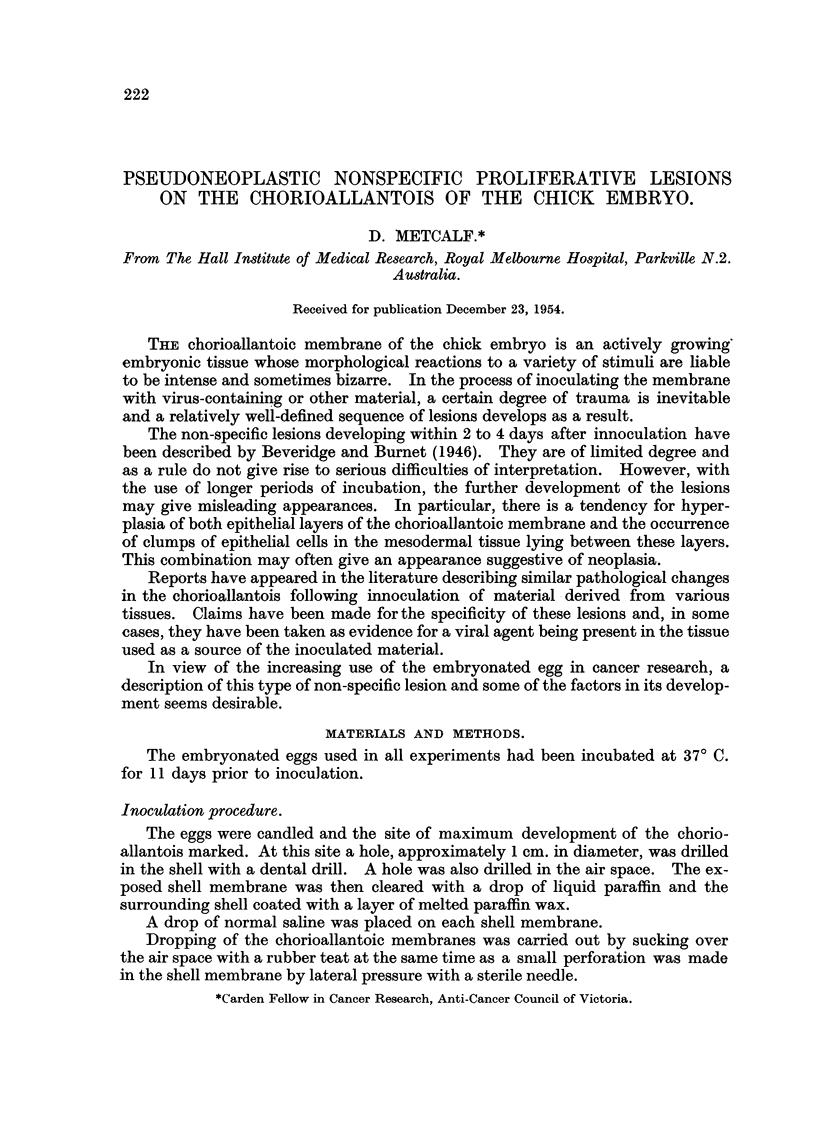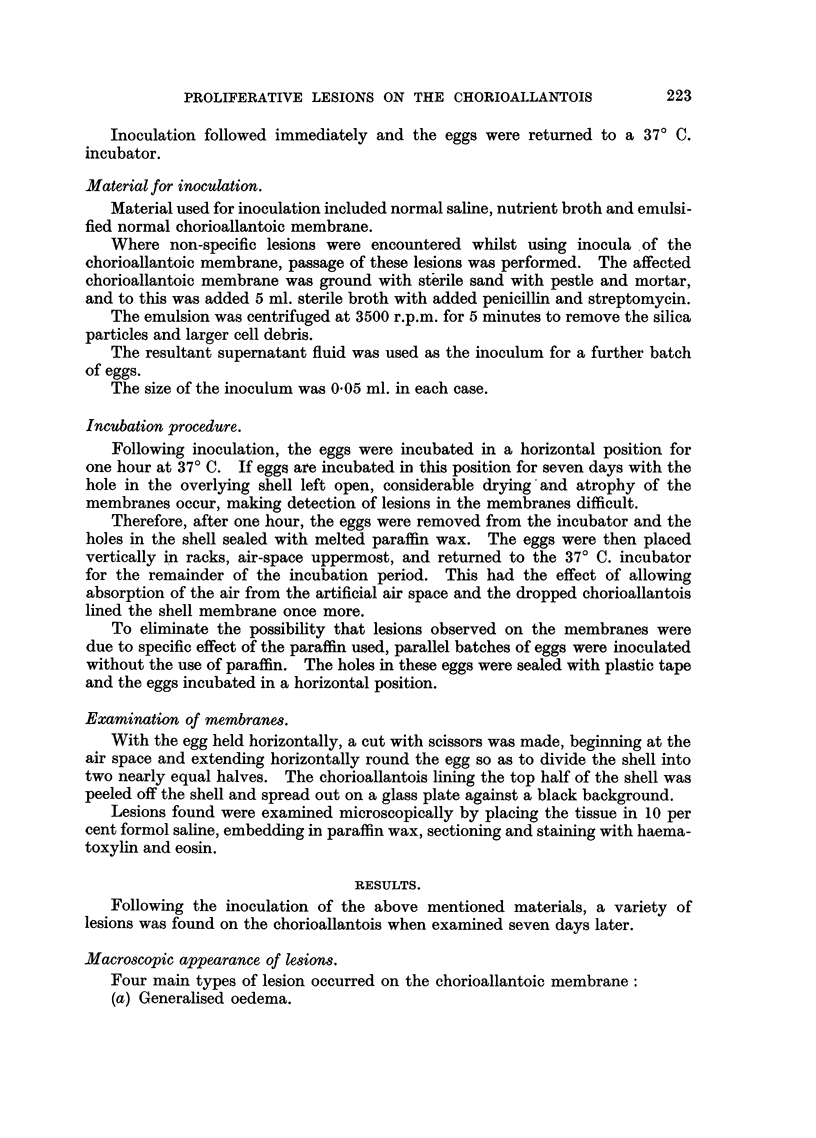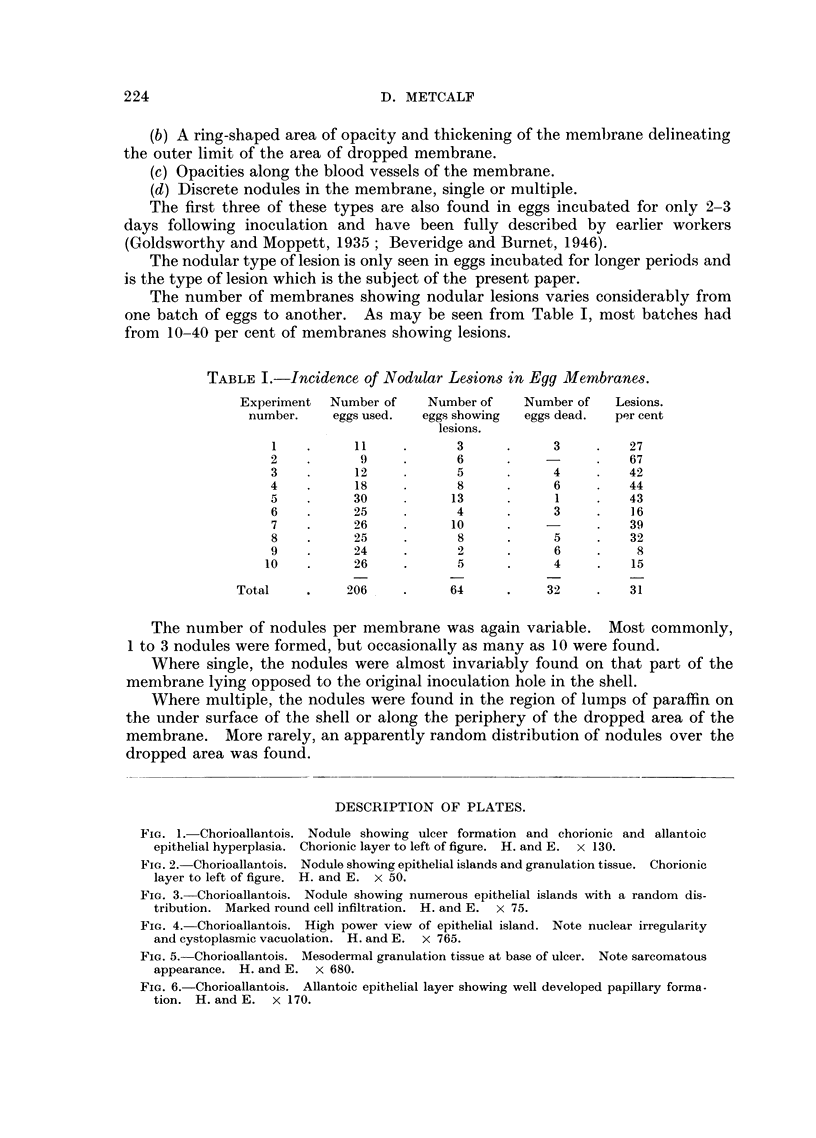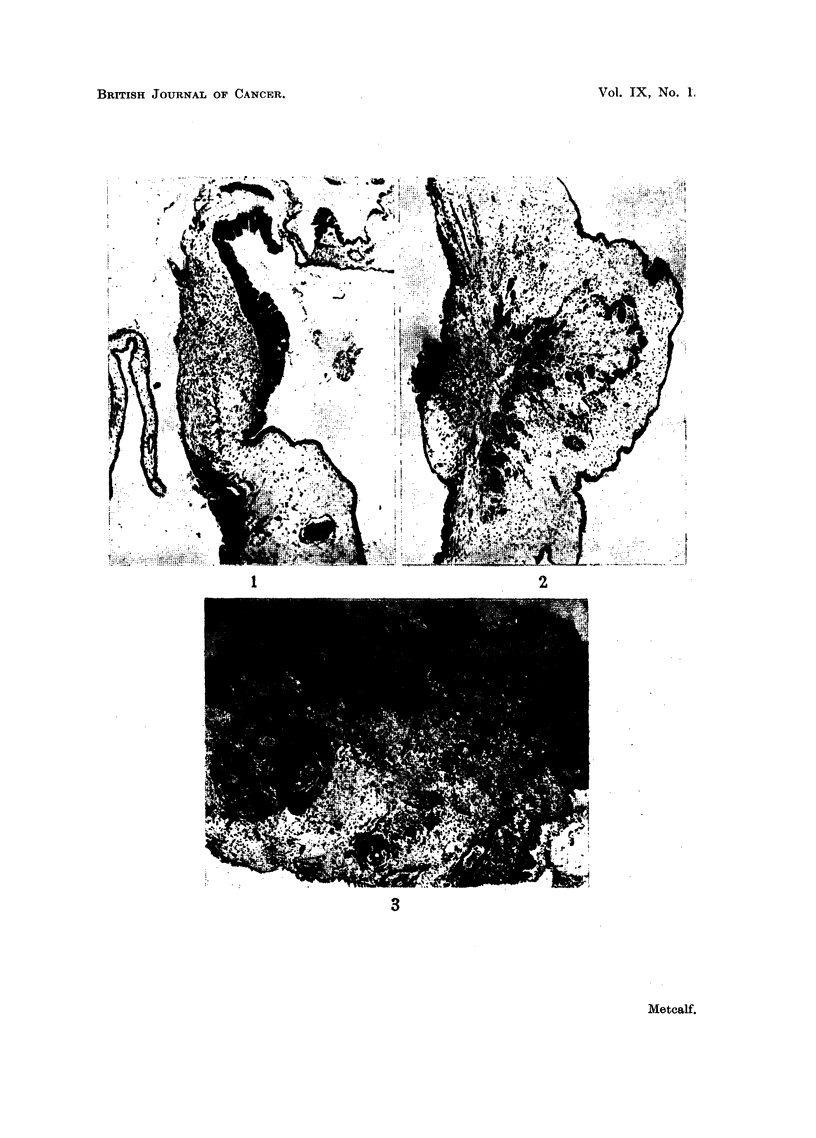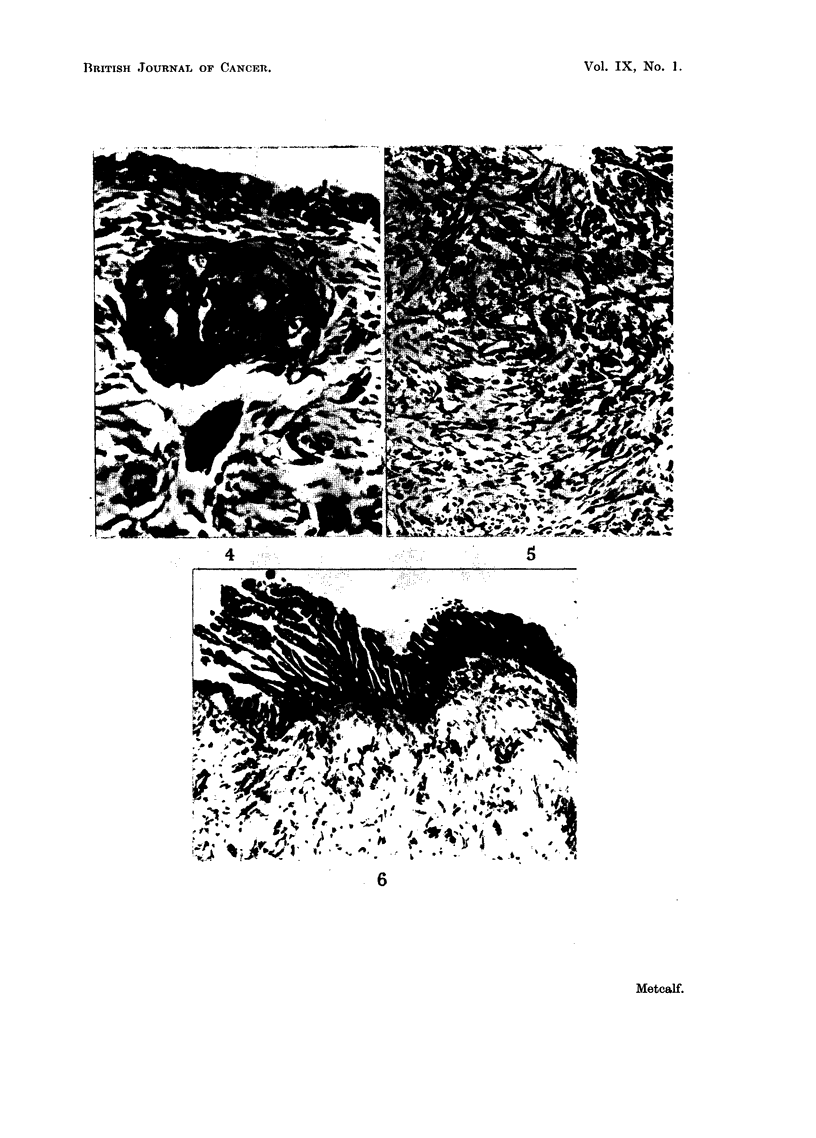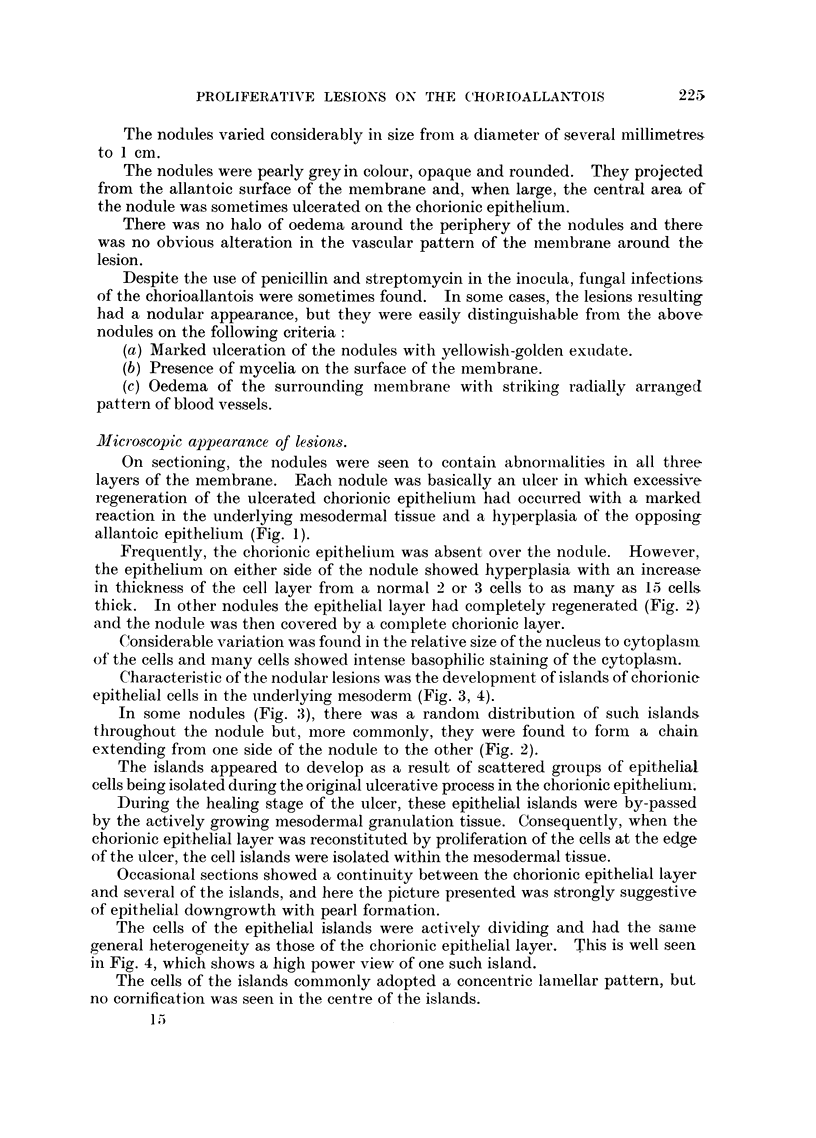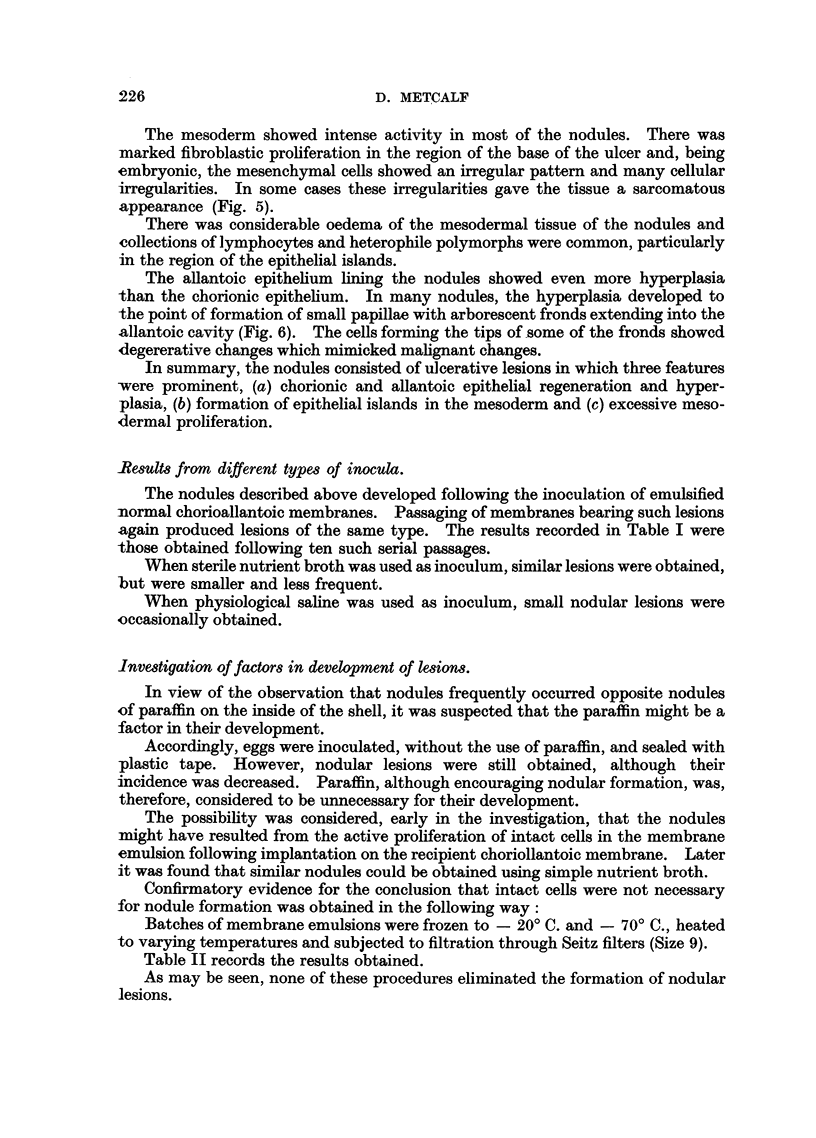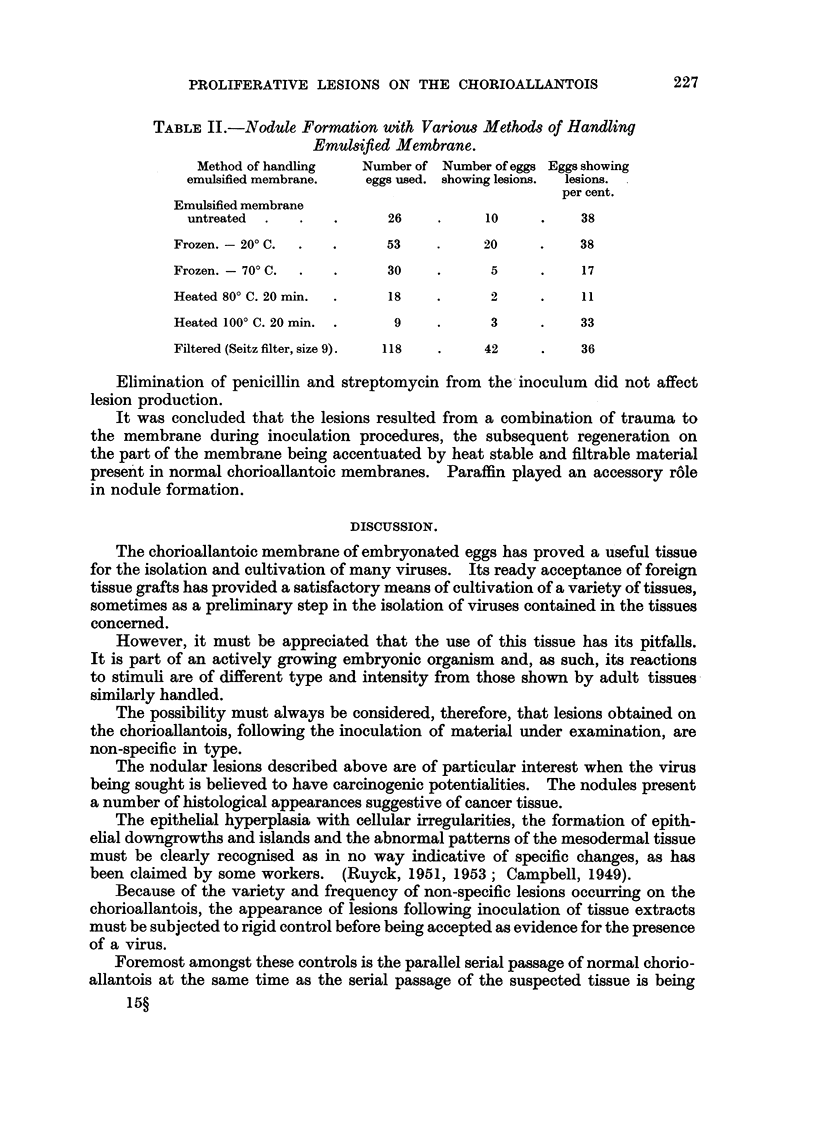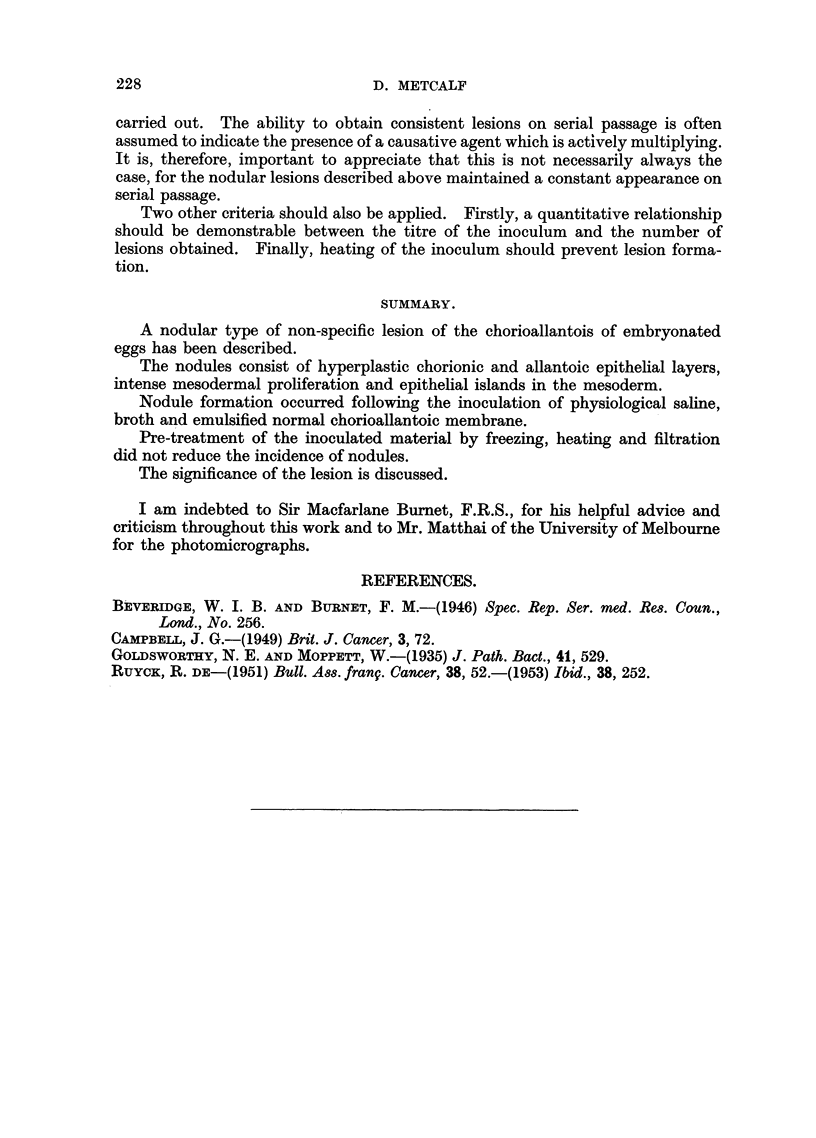# Pseudoneoplastic Nonspecific Proliferative Lesions on the Chorioallantois of the Chick Embryo

**DOI:** 10.1038/bjc.1955.18

**Published:** 1955-03

**Authors:** D. Metcalf

## Abstract

**Images:**


### PSEUDONEOPLASTIC NONSPECIFIC PROLIFERATIVE LESIONS ON THE CHORIOALLANTOIS OF THE CHICK EMBRYO.

D. METCALF.*

*From The Hall Institute of Medical Research, Royal Melbourne Hospital, Parkville N.2. Australia.*

Received for publication December 23, 1954.

THE chorioallantoic membrane of the chick embryo is an actively growing embryonic tissue whose morphological reactions to a variety of stimuli are liable to be intense and sometimes bizarre. In the process of inoculating the membrane with virus-containing or other material, a certain degree of trauma is inevitable and a relatively well-defined sequence of lesions develops as a result.

The non-specific lesions developing within 2 to 4 days after innoculation have been described by Beveridge and Burnet (1946). They are of limited degree and as a rule do not give rise to serious difficulties of interpretation. However, with the use of longer periods of incubation, the further development of the lesions may give misleading appearances. In particular, there is a tendency for hyperplasia of both epithelial layers of the chorioallantoic membrane and the occurrence of clumps of epithelial cells in the mesodermal tissue lying between these layers. This combination may often give an appearance suggestive of neoplasia.

Reports have appeared in the literature describing similar pathological changes in the chorioallantois following innoculation of material derived from various tissues. Claims have been made for the specificity of these lesions and, in some cases, they have been taken as evidence for a viral agent being present in the tissue used as a source of the inoculated material.

In view of the increasing use of the embryonated egg in cancer research, a description of this type of non-specific lesion and some of the factors in its development seems desirable.

### MATERIALS AND METHODS.

The embryonated eggs used in all experiments had been incubated at 37° C. for 11 days prior to inoculation.

### *Inoculation procedure.*

The eggs were candled and the site of maximum development of the chorioallantois marked. At this site a hole, approximately 1 cm. in diameter, was drilled in the shell with a dental drill. A hole was also drilled in the air space. The exposed shell membrane was then cleared with a drop of liquid paraffin and the surrounding shell coated with a layer of melted paraffin wax.

A drop of normal saline was placed on each shell membrane.

Dropping of the chorioallantoic membranes was carried out by sucking over the air space with a rubber teat at the same time as a small perforation was made in the shell membrane by lateral pressure with a sterile needle.

*Carden Fellow in Cancer Research, Anti-Cancer Council of Victoria.

Inoculation followed immediately and the eggs were returned to a 37° C. incubator.

*Material for inoculation.*

Material used for inoculation included normal saline, nutrient broth and emulsified normal chorioallantoic membrane.

Where non-specific lesions were encountered whilst using inocula of the chorioallantoic membrane, passage of these lesions was performed. The affected chorioallantoic membrane was ground with sterile sand with pestle and mortar, and to this was added 5 ml. sterile broth with added penicillin and streptomycin.

The emulsion was centrifuged at 3500 r.p.m. for 5 minutes to remove the silica particles and larger cell debris.

The resultant supernatant fluid was used as the inoculum for a further batch of eggs.

The size of the inoculum was 0·05 ml. in each case.

*Incubation procedure.*

Following inoculation, the eggs were incubated in a horizontal position for one hour at 37° C. If eggs are incubated in this position for seven days with the hole in the overlying shell left open, considerable drying and atrophy of the membranes occur, making detection of lesions in the membranes difficult.

Therefore, after one hour, the eggs were removed from the incubator and the holes in the shell sealed with melted paraffin wax. The eggs were then placed vertically in racks, air-space uppermost, and returned to the 37° C. incubator for the remainder of the incubation period. This had the effect of allowing absorption of the air from the artificial air space and the dropped chorioallantois lined the shell membrane once more.

To eliminate the possibility that lesions observed on the membranes were due to specific effect of the paraffin used, parallel batches of eggs were inoculated without the use of paraffin. The holes in these eggs were sealed with plastic tape and the eggs incubated in a horizontal position.

*Examination of membranes.*

With the egg held horizontally, a cut with scissors was made, beginning at the air space and extending horizontally round the egg so as to divide the shell into two nearly equal halves. The chorioallantois lining the top half of the shell was peeled off the shell and spread out on a glass plate against a black background.

Lesions found were examined microscopically by placing the tissue in 10 per cent formol saline, embedding in paraffin wax, sectioning and staining with haematoxylin and eosin.

### RESULTS.

Following the inoculation of the above mentioned materials, a variety of lesions was found on the chorioallantois when examined seven days later.

*Macroscopic appearance of lesions.*

Four main types of lesion occurred on the chorioallantoic membrane :

(*a*) Generalised oedema.

(*b*) A ring-shaped area of opacity and thickening of the membrane delineating the outer limit of the area of dropped membrane.

(*c*) Opacities along the blood vessels of the membrane.

(*d*) Discrete nodules in the membrane, single or multiple.

The first three of these types are also found in eggs incubated for only 2–3 days following inoculation and have been fully described by earlier workers (Goldsworthy and Moppett, 1935 ; Beveridge and Burnet, 1946).

The nodular type of lesion is only seen in eggs incubated for longer periods and is the type of lesion which is the subject of the present paper.

The number of membranes showing nodular lesions varies considerably from one batch of eggs to another. As may be seen from Table I, most batches had from 10–40 per cent of membranes showing lesions.

TABLE I.—*Incidence of Nodular Lesions in Egg Membranes.*

| Experiment number. | Number of eggs used. | Number of eggs showing lesions. | Number of eggs dead. | Lesions. per cent |
|---|---|---|---|---|
| 1 | 11 | 3 | 3 | 27 |
| 2 | 9 | 6 | — | 67 |
| 3 | 12 | 5 | 4 | 42 |
| 4 | 18 | 8 | 6 | 44 |
| 5 | 30 | 13 | 1 | 43 |
| 6 | 25 | 4 | 3 | 16 |
| 7 | 26 | 10 | — | 39 |
| 8 | 25 | 8 | 5 | 32 |
| 9 | 24 | 2 | 6 | 8 |
| 10 | 26 | 5 | 4 | 15 |
| Total | 206 | 64 | 32 | 31 |

The number of nodules per membrane was again variable. Most commonly, 1 to 3 nodules were formed, but occasionally as many as 10 were found.

Where single, the nodules were almost invariably found on that part of the membrane lying opposed to the original inoculation hole in the shell.

Where multiple, the nodules were found in the region of lumps of paraffin on the under surface of the shell or along the periphery of the dropped area of the membrane. More rarely, an apparently random distribution of nodules over the dropped area was found.

### DESCRIPTION OF PLATES.

FIG. 1.—Chorioallantois. Nodule showing ulcer formation and chorionic and allantoic epithelial hyperplasia. Chorionic layer to left of figure. H. and E. × 130.

FIG. 2.—Chorioallantois. Nodule showing epithelial islands and granulation tissue. Chorionic layer to left of figure. H. and E. × 50.

FIG. 3.—Chorioallantois. Nodule showing numerous epithelial islands with a random distribution. Marked round cell infiltration. H. and E. × 75.

FIG. 4.—Chorioallantois. High power view of epithelial island. Note nuclear irregularity and cystoplasmic vacuolation. H. and E. × 765.

FIG. 5.—Chorioallantois. Mesodermal granulation tissue at base of ulcer. Note sarcomatous appearance. H. and E. × 680.

FIG. 6.—Chorioallantois. Allantoic epithelial layer showing well developed papillary formation. H. and E. × 170.

1

2

3

4 5

6

Metcalf.

The nodules varied considerably in size from a diameter of several millimetres to 1 cm.

The nodules were pearly grey in colour, opaque and rounded. They projected from the allantoic surface of the membrane and, when large, the central area of the nodule was sometimes ulcerated on the chorionic epithelium.

There was no halo of oedema around the periphery of the nodules and there was no obvious alteration in the vascular pattern of the membrane around the lesion.

Despite the use of penicillin and streptomycin in the inocula, fungal infections of the chorioallantois were sometimes found. In some cases, the lesions resulting had a nodular appearance, but they were easily distinguishable from the above nodules on the following criteria :

(*a*) Marked ulceration of the nodules with yellowish-golden exudate.

(*b*) Presence of mycelia on the surface of the membrane.

(*c*) Oedema of the surrounding membrane with striking radially arranged pattern of blood vessels.

*Microscopic appearance of lesions.*

On sectioning, the nodules were seen to contain abnormalities in all three layers of the membrane. Each nodule was basically an ulcer in which excessive regeneration of the ulcerated chorionic epithelium had occurred with a marked reaction in the underlying mesodermal tissue and a hyperplasia of the opposing allantoic epithelium (Fig. 1).

Frequently, the chorionic epithelium was absent over the nodule. However, the epithelium on either side of the nodule showed hyperplasia with an increase in thickness of the cell layer from a normal 2 or 3 cells to as many as 15 cells thick. In other nodules the epithelial layer had completely regenerated (Fig. 2) and the nodule was then covered by a complete chorionic layer.

Considerable variation was found in the relative size of the nucleus to cytoplasm of the cells and many cells showed intense basophilic staining of the cytoplasm.

Characteristic of the nodular lesions was the development of islands of chorionic epithelial cells in the underlying mesoderm (Fig. 3, 4).

In some nodules (Fig. 3), there was a random distribution of such islands throughout the nodule but, more commonly, they were found to form a chain extending from one side of the nodule to the other (Fig. 2).

The islands appeared to develop as a result of scattered groups of epithelial cells being isolated during the original ulcerative process in the chorionic epithelium.

During the healing stage of the ulcer, these epithelial islands were by-passed by the actively growing mesodermal granulation tissue. Consequently, when the chorionic epithelial layer was reconstituted by proliferation of the cells at the edge of the ulcer, the cell islands were isolated within the mesodermal tissue.

Occasional sections showed a continuity between the chorionic epithelial layer and several of the islands, and here the picture presented was strongly suggestive of epithelial downgrowth with pearl formation.

The cells of the epithelial islands were actively dividing and had the same general heterogeneity as those of the chorionic epithelial layer. This is well seen in Fig. 4, which shows a high power view of one such island.

The cells of the islands commonly adopted a concentric lamellar pattern, but no cornification was seen in the centre of the islands.

The mesoderm showed intense activity in most of the nodules. There was marked fibroblastic proliferation in the region of the base of the ulcer and, being embryonic, the mesenchymal cells showed an irregular pattern and many cellular irregularities. In some cases these irregularities gave the tissue a sarcomatous appearance (Fig. 5).

There was considerable oedema of the mesodermal tissue of the nodules and collections of lymphocytes and heterophile polymorphs were common, particularly in the region of the epithelial islands.

The allantoic epithelium lining the nodules showed even more hyperplasia than the chorionic epithelium. In many nodules, the hyperplasia developed to the point of formation of small papillae with arborescent fronds extending into the allantoic cavity (Fig. 6). The cells forming the tips of some of the fronds showcd degererative changes which mimicked malignant changes.

In summary, the nodules consisted of ulcerative lesions in which three features were prominent, (*a*) chorionic and allantoic epithelial regeneration and hyperplasia, (*b*) formation of epithelial islands in the mesoderm and (*c*) excessive mesodermal proliferation.

*Results from different types of inocula.*

The nodules described above developed following the inoculation of emulsified normal chorioallantoic membranes. Passaging of membranes bearing such lesions again produced lesions of the same type. The results recorded in Table I were those obtained following ten such serial passages.

When sterile nutrient broth was used as inoculum, similar lesions were obtained, but were smaller and less frequent.

When physiological saline was used as inoculum, small nodular lesions were occasionally obtained.

*Investigation of factors in development of lesions.*

In view of the observation that nodules frequently occurred opposite nodules of paraffin on the inside of the shell, it was suspected that the paraffin might be a factor in their development.

Accordingly, eggs were inoculated, without the use of paraffin, and sealed with plastic tape. However, nodular lesions were still obtained, although their incidence was decreased. Paraffin, although encouraging nodular formation, was, therefore, considered to be unnecessary for their development.

The possibility was considered, early in the investigation, that the nodules might have resulted from the active proliferation of intact cells in the membrane emulsion following implantation on the recipient choriollantoic membrane. Later it was found that similar nodules could be obtained using simple nutrient broth.

Confirmatory evidence for the conclusion that intact cells were not necessary for nodule formation was obtained in the following way :

Batches of membrane emulsions were frozen to — 20° C. and — 70° C., heated to varying temperatures and subjected to filtration through Seitz filters (Size 9).

Table II records the results obtained.

As may be seen, none of these procedures eliminated the formation of nodular lesions.

TABLE II.—*Nodule Formation with Various Methods of Handling Emulsified Membrane.*

| Method of handling emulsified membrane. | Number of eggs used. | Number of eggs showing lesions. | Eggs showing lesions. per cent. |
|---|---|---|---|
| Emulsified membrane untreated | 26 | 10 | 38 |
| Frozen. – 20° C. | 53 | 20 | 38 |
| Frozen. – 70° C. | 30 | 5 | 17 |
| Heated 80° C. 20 min. | 18 | 2 | 11 |
| Heated 100° C. 20 min. | 9 | 3 | 33 |
| Filtered (Seitz filter, size 9). | 118 | 42 | 36 |

Elimination of penicillin and streptomycin from the inoculum did not affect lesion production.

It was concluded that the lesions resulted from a combination of trauma to the membrane during inoculation procedures, the subsequent regeneration on the part of the membrane being accentuated by heat stable and filtrable material present in normal chorioallantoic membranes. Paraffin played an accessory rôle in nodule formation.

### DISCUSSION.

The chorioallantoic membrane of embryonated eggs has proved a useful tissue for the isolation and cultivation of many viruses. Its ready acceptance of foreign tissue grafts has provided a satisfactory means of cultivation of a variety of tissues, sometimes as a preliminary step in the isolation of viruses contained in the tissues concerned.

However, it must be appreciated that the use of this tissue has its pitfalls. It is part of an actively growing embryonic organism and, as such, its reactions to stimuli are of different type and intensity from those shown by adult tissues similarly handled.

The possibility must always be considered, therefore, that lesions obtained on the chorioallantois, following the inoculation of material under examination, are non-specific in type.

The nodular lesions described above are of particular interest when the virus being sought is believed to have carcinogenic potentialities. The nodules present a number of histological appearances suggestive of cancer tissue.

The epithelial hyperplasia with cellular irregularities, the formation of epithelial downgrowths and islands and the abnormal patterns of the mesodermal tissue must be clearly recognised as in no way indicative of specific changes, as has been claimed by some workers. (Ruyck, 1951, 1953 ; Campbell, 1949).

Because of the variety and frequency of non-specific lesions occurring on the chorioallantois, the appearance of lesions following inoculation of tissue extracts must be subjected to rigid control before being accepted as evidence for the presence of a virus.

Foremost amongst these controls is the parallel serial passage of normal chorioallantois at the same time as the serial passage of the suspected tissue is being carried out. The ability to obtain consistent lesions on serial passage is often assumed to indicate the presence of a causative agent which is actively multiplying. It is, therefore, important to appreciate that this is not necessarily always the case, for the nodular lesions described above maintained a constant appearance on serial passage.

Two other criteria should also be applied. Firstly, a quantitative relationship should be demonstrable between the titre of the inoculum and the number of lesions obtained. Finally, heating of the inoculum should prevent lesion formation.

SUMMARY.

A nodular type of non-specific lesion of the chorioallantois of embryonated eggs has been described.

The nodules consist of hyperplastic chorionic and allantoic epithelial layers, intense mesodermal proliferation and epithelial islands in the mesoderm.

Nodule formation occurred following the inoculation of physiological saline, broth and emulsified normal chorioallantoic membrane.

Pre-treatment of the inoculated material by freezing, heating and filtration did not reduce the incidence of nodules.

The significance of the lesion is discussed.

I am indebted to Sir Macfarlane Burnet, F.R.S., for his helpful advice and criticism throughout this work and to Mr. Matthai of the University of Melbourne for the photomicrographs.

### REFERENCES.

Beveridge, W. I. B. and Burnet, F. M.—(1946) *Spec. Rep. Ser. med. Res. Coun., Lond., No.* 256.

Campbell, J. G.—(1949) *Brit. J. Cancer*, **3**, 72.

Goldsworthy, N. E. and Moppett, W.—(1935) *J. Path. Bact.*, **41**, 529.

Ruyck, R. de—(1951) *Bull. Ass. franç. Cancer*, **38**, 52.—(1953) *Ibid.*, **38**, 252.